# Chest wall mass as a sign of ignored hepatocellular carcinoma in an alcoholic cirrhotic patient: a case report

**DOI:** 10.1097/MS9.0000000000001007

**Published:** 2023-06-20

**Authors:** Elisha Poddar, Suraj Shrestha, Raju Thapa, Sudhan Subedi, Niharika Pathak, Ramesh Khadayat, Pradeep Regmi

**Affiliations:** a Maharajgunj Medical Campus, Institute of Medicine; Departments of b Surgical Gastroenterology; c Radiology, Tribhuvan University Teaching Hospital, Kathmandu, Nepal

**Keywords:** bone metastasis, hepatocellular carcinoma, prognosis, case report

## Abstract

Bone metastasis of hepatocellular carcinoma (HCC) is usually seen in the course of advanced intrahepatic HCC. Isolated bone metastasis as an initial manifestation of HCC is uncommon.

**Case presentation:**

The authors report a case of a 68-year-old male with a history of chronic alcohol consumption who presented with epigastric pain, abdominal distension, and a hard, tender lump on the right posterolateral aspect of his back. Investigation revealed HCC with isolated metastasis to the posterior thoracic wall.

**Clinical discussion:**

HCC usually develops secondary to chronic hepatitis B and C infection in the background of chronic liver disease. Common presenting symptoms of bone metastasis include local pain, neurological manifestations, palpable subcutaneous masses, and pathological fractures. An immunohistochemistry analysis is important to differentiate HCC from non-HCC metastasis in patients without known underlying HCC. Treatment is often directed towards palliative care as the prognosis is poor.

**Conclusion:**

An isolated rib mass can be an initial presentation of metastatic HCC. Thus, HCC with bone metastasis should be considered in the differential diagnosis in patients presenting with painful swelling in the thoracic region.

## Introduction

Highlights

- Bone metastasis is often a feature of an advanced hepatocellular carcinoma (HCC).
- Isolated bony mass as an initial presentation of metastatic HCC is exceedingly uncommon.
- Metastatic HCC can be a differential diagnosis in painful swelling in the thoracic region.

Hepatocellular carcinoma (HCC) is the seventh most common cancer, accounting for ~75% of the total primary liver cancer and the second leading cause of cancer mortality worldwide^1,2^. The clinical presentations of patients with HCC are mostly concerned with the manifestations of the primary tumour and a metastatic presentation is a later event^3^. Even in patients with advanced HCC, the rate of extrahepatic metastasis is limited^4^. The lung, abdominal lymph nodes, bone, and adrenals are the most common sites of extrahepatic metastasis^5–7^. Bone metastasis typically involves the spine, pelvis, ribs, and skull, has an extremely poor prognosis, and infrequently be the primary manifestation of HCC^8^.

The initial manifestation of unsuspected HCC as a bone metastasis is rare^9^. In a series of 395 patients with pathologically verified hepatocellular carcinoma, 20 patients (5%) had bone metastasis at initial presentation^10^. Malignant chest wall tumours, especially those metastasized from HCCs are uncommon as the chest wall is a rare site of HCC metastasis. In a report by Katyal *et al.* ^11^, only 6 of 148 HCC patients had extrahepatic lesions in the chest wall. In addition, isolated metastases of HCC to the ribs have been seldom reported^12–14^.

We report a case of a 68-year-old man who presented with a tender lump over his right posterior rib, which revealed metastatic carcinoma with HCC of the liver. This case has been reported in line with SCARE criteria^15^.

## Case presentation

**Figure 1:**
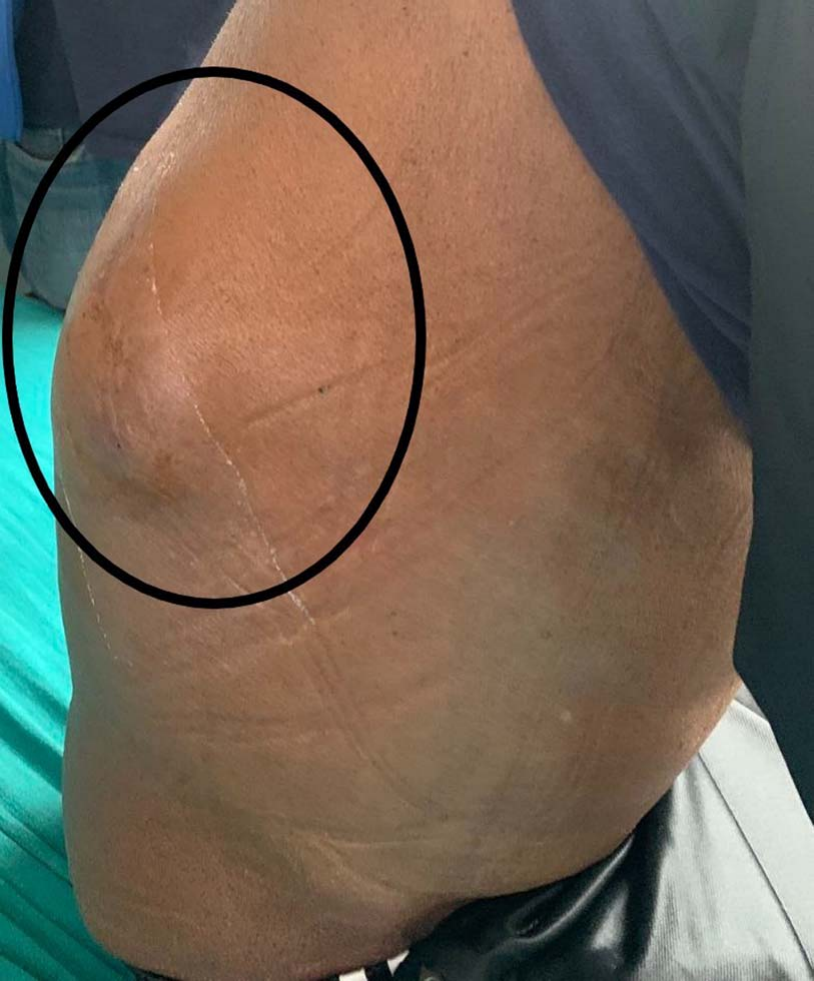
Lump over the right side of the back.

**Figure 2:**
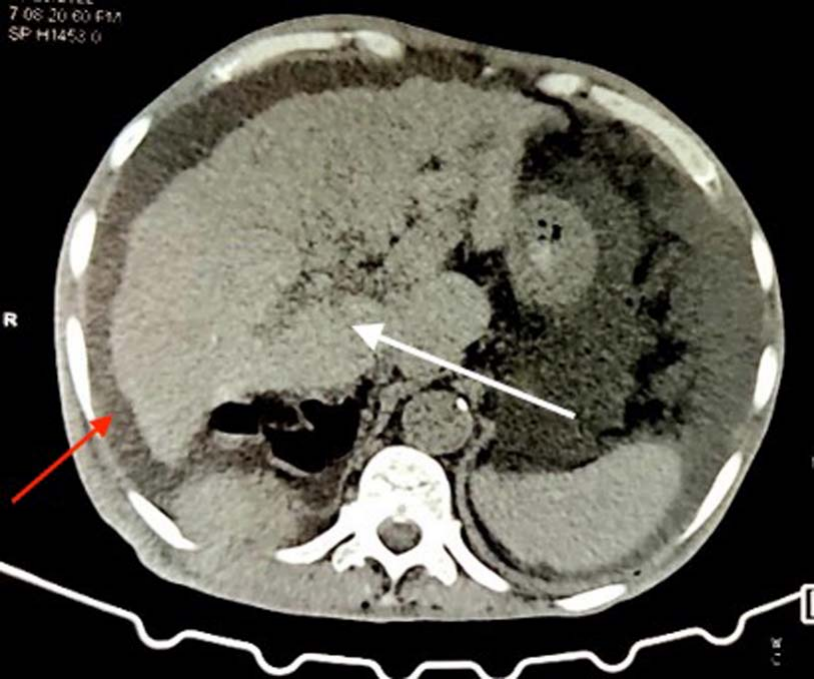
Axial contrast-enhanced abdomen CT shows diffuse irregularity of liver (white arrow) and ascites (red arrow). CT, computed tomography.

**Figure 3:**
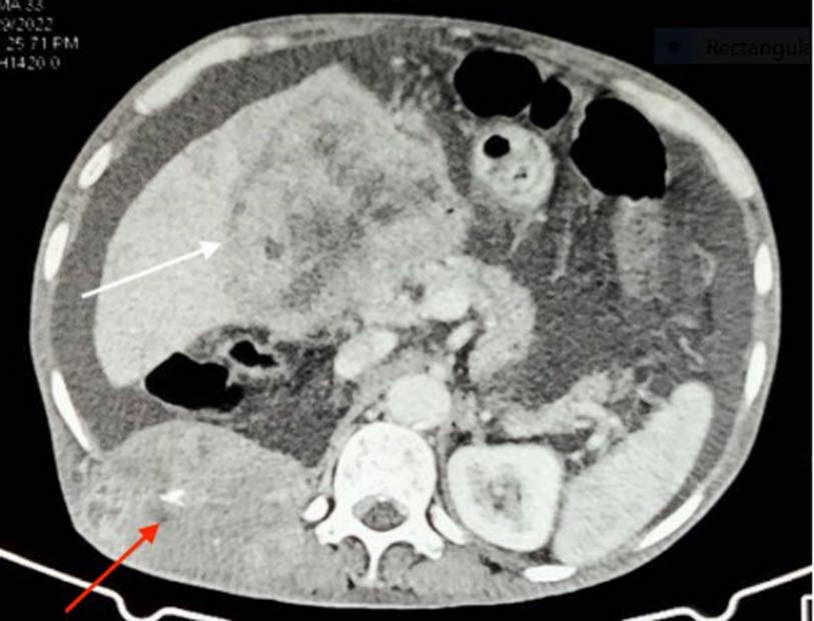
Axial contrast-enhanced abdomen CT shows well-defined heterogeneously enhancing liver mass (white arrow) and enhancing soft tissue density along with erosion of posterior rib (red arrow). CT, computed tomography.

**Figure 4:**
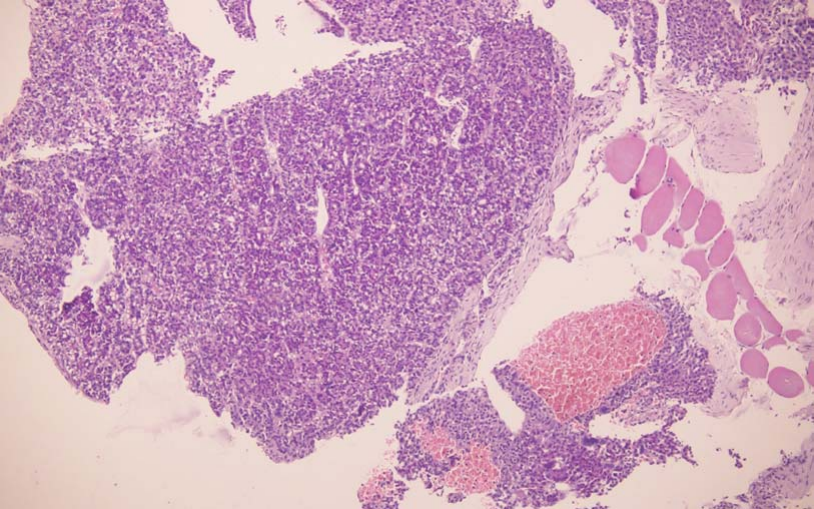
A biopsy of the right thoracic mass revealed tumour cells with a high nuclear-cytoplasmic ratio, arranged in solid sheets and acinus.

A 68-year-old man from a remote part of Nepal, who used to consume alcohol, presented with a slowly growing tender lump on the lateral aspect of his right back for 2 months. Additionally, he experienced progressive abdominal distension, epigastric pain, and swelling in his lower limbs for one and a half months, accompanied by significant weight loss. Despite his history of consuming home-brewed alcohol of about one liter per day for almost 30 years, he denied any previous symptoms suggestive chronic liver disease. He did not report any yellowish discoloration of his body parts, alterations in bowel habits, or frothy urine before the onset of these symptoms. Furthermore, there was no history of malignancy in his family or personal medical history. On examination, he appeared icteric and had a firm enlarged with tense ascites. Additionally, he had bilateral lower limb pitting oedema. Upon further inspection, an ~8×5 cm hard, tender, and immobile mass spanning the right lower posterior ribs was found (Fig. 1). The rest of the examination was unremarkable. Blood investigation results showed a deranged liver function test and non-reactive serology. An ultrasonography scan of the abdomen and pelvis revealed hepatomegaly with an irregular outline and coarse echotexture with heteroechoic mass in the right lobe of the liver along with ascites. The patient’s serum alpha-fetoprotein (AFP) level was remarkably elevated, measuring above 2000 ng/ml (Normal range: 0–8.78 ng/ml). The ascitic fluid analysis was negative for malignant cytology. A contrast-enhanced computed tomography scan of the chest, abdomen, and pelvis showed an ~12.5×12.1×7.7 cm exophytic, ill-defined, irregular, heterogeneous soft tissue density arising from the inferior surface of the liver with heterogeneous enhancement in post-contrast images with compression of the main and left portal vein (Fig. 2). Additionally, ~10.2×6.5 cm heterogeneously enhancing soft tissue density in the right posterior chest wall causing erosion of the 11th posterior rib along with the involvement of all muscle layers and subcutaneous tissue and gross ascites with minimal pleural effusion was observed; features suggestive of hepatocellular carcinoma with portal vein thrombosis with bony metastasis in the right posterior chest. No mass lesions were noted elsewhere. (Fig. 3) A biopsy of the right thoracic mass revealed tumour cells with a high nuclear-to-cytoplasmic ratio, arranged in solid sheets and acinus suggestive of metastatic carcinoma. (Fig. 4) As the patient could not afford further investigations, an immunohistochemical analysis could not be performed. With a diagnosis of HCC with bony metastasis, the patient was given comprehensive counselling regarding the prognosis and treatment options, including radiotherapy and the chemotherapeutic agent sorafenib. Despite this, due to financial constraints, the patient declined further treatment and was instead managed palliatively with painkillers. The patient was discharged upon request and was subsequently lost to follow-up.

## Discussion

HCC usually develops secondary to chronic hepatitis B and C infection in the background of chronic liver disease^16^. Our patient did not have any chronic viral infections, however, was a chronic alcohol drinker without any prior investigations. Patients presenting with the extrahepatic disease generally have advanced tumours (>5 cm, multifocal, with vascular invasion)^17^. Although bone metastasis may occur in around 10% of cases with HCC, it is often seen with advanced intrahepatic HCC^18^. Bony metastases in HCC are generally multiple; isolated bony metastasis as an initial presentation, as in our case, is very rare.

Skeletal metastasis is unique among various hematogenous metastases of HCC as it can occur before clinical manifestations of liver disease and is usually symptomatic, as in the present case. Common presenting symptoms include local pain, neurological manifestations, palpable subcutaneous masses, and pathological fractures^19^.

In metastasis, differentiation of HCC from non-HCC may cause a diagnostic problem as histologic patterns of HCC mimic a wide variety of malignant tumours and vice-versa^20,21^.

In patients with a history of primary HCC, it is easy to consider the metastasis as HCC in comparison with patients without any manifestation of HCC, especially in poorly differentiated tumours^20^. Yan *et al.* ^22^ suggested if the patient has a known primary and the aspirate demonstrates malignant cells, comparing the FNA sample with available histologic or cytologic slides from the original tumour can help establish a definitive diagnosis. Despite all, immunohistochemistry is confirmatory. An immunohistochemical panel including ARG-1, HepPar-1, AFP and GPC-3, TTF-1, napsin-A, GATA3, CDX2, PAX5, PSA can be useful in differentiating HCC and non-HCC in most metastatic cases^20,21^. Timek and colleagues recommended using a panel of three markers that is ARG-1, GPC-3, HepPar-1, and AFP, which has high sensitivity and specificity^20,21^. In view of raised AFP, computed tomography scan features consistent with HCC, absence of other malignant lesions, and histopathology of the rib lesion consistent with metastatic carcinoma, a diagnosis of bony rib metastasis with HCC was made in our patient.

Patients with HCC who have developed bone metastasis (HCC-BM) have a poor prognosis and a median survival time of only around 4.6 months^23^. Existing guidelines approve targeted therapy, systemic chemotherapy, or best supportive care for HCC patients with extrahepatic metastasis. In addition, bone-directed local radiation like external beam radiation can be used as a palliative treatment for the management of painful bony metastasis but overall survival with radiation therapy is poor^24–26^.

The multikinase inhibitors sorafenib and lenvatinib are the approved first-line systemic treatments for unresectable hepatocellular carcinoma. However, both are associated with considerable side effects hampering the quality of life^27,28^.

The combination of atezolizumab and bevacizumab is a promising immunotherapy combination with targeted therapy^29^. Treatment with atezolizumab plus bevacizumab was associated with significantly better overall survival and progression-free survival outcomes than sorafenib in patients with advanced unresectable hepatocellular carcinoma^30^. Majorities of HCC patients with extrahepatic metastasis died of progressive intrahepatic tumours leading to hepatic failure, but not extrahepatic metastases, and primary tumour resection may have a favourable impact on the prognosis of these patients with resectable primary tumours. However, in patients with extrahepatic metastasis, the role of resection of metastatic tumours remains unclear^31^. In our case, he was counselled regarding treatment options; however, he denied further treatment considering the financial status of the family and grave prognosis.

## Conclusion

An isolated rib mass can be an initial presentation of metastatic HCC. Thus, HCC with bone metastasis should be considered in the differential diagnosis in patients presenting with painful swelling in the thoracic region.

## Ethical approval

Not applicable as ethical approval is not required for writing the case report from the institutional review board in our institute.

## Consent

Written informed consent was obtained from the patient’s party for publication of this case report and accompanying images. A copy of the written consent is available for review by the Editor-in-Chief of this journal on request.

## Source of funding

This research work did not receive any kind of funding.

## Author contribution

E.P.: conceptualization, resources, data curation, writing—original draft, writing—review and editing, supervision, project administration. S.S.: conceptualization, resources, data curation, writing—original draft, writing—review and editing. R.T. and S.S.: conceptualization, resources, writing—review and editing. N.P. and R.K.: writing—original draft, writing—review and editing. P.R.: writing—original draft, writing—review and editing, supervision.

## Conflicts of interest disclosure

None to declare.

## Research registration unique identifying number (UIN)

Not applicable.

## Guarantor

Dr. Suraj Shrestha.

## Data availability statement

All the necessary information is provided within the manuscript.

## Provenance and peer review

Not commissioned, externally peer-reviewed.
